# Multiplex PCR for simultaneous identification of *Ralstonia solanacearum* and *Xanthomonas perforans*

**DOI:** 10.1007/s13205-014-0223-z

**Published:** 2014-05-17

**Authors:** S. Umesha, P. Avinash

**Affiliations:** Department of Studies in Biotechnology, University of Mysore, Manasagangotri, Mysore, 570006 Karnataka India

**Keywords:** Multiplex PCR, *Ralstonia solanacearum*, *Xanthomonas perforans*, Bacterial wilt, Bacterial spot-tomato

## Abstract

*Ralstonia solanacearum* is a causative agent of bacterial wilt in many economically important crops, and *Xanthomonas perforans* is the causal organism of bacterial spot, one of the most important diseases of vegetables. A multiplex PCR protocol has been developed for the simultaneous, specific and rapid identification of *R. solanacearum* and *X. perforans* in plant materials. Species-specific primers RS-F-759 and RS-R-760 for *R. solanacearum,* RST2 and RST3 for *X. perforans* were used for identification of both pathogens at primer concentrations of 1:4 by optimization of multiplex PCR at annealing temperature of about 61 ± 1 °C. With these primer sets, specific amplification of 281- and 840-bp PCR products was obtained for *R. solanacearum* and *X. perforans*, respectively. The multiplex PCR assay was validated with susceptible plants mechanically inoculated with both the pathogens; specific PCR products confirmed the presence of *R. solanacearum* and *X. perforans.* The multiplex PCR is valuable in identification as well as primary screening of cultivars of both pathogens. The present study is a rapid and easy method for early identification of pathogens from asymptomatic and symptomatic plant materials.

## Introduction

Globalization of world agriculture increases easy movement of infectious plant material across the countries, which leads to severe problems and difficulty in controlling the spread of plant pathogens, and set back in world agricultural economy by severe yield losses. Robust and inexpensive diagnostic tools are not available for easy identification and classification of many plant pathogens. The primary hurdle in developing highly specific, easily usable diagnostic tools for any pathogen has been difficult in finding unique features, whether they are cell surface antigens or DNA sequences. Therefore, there is a principal need to develop reliable, fast and specific identification methods to prevent spread of diseases caused by several phytopathogenic bacteria.

*Ralstonia solanacearum* causes bacterial wilt, which is one of the most important and widely spread bacterial diseases of Solanaceous crops in tropics, subtropics and warm temperate regions of the world. This disease has also been recorded in more than 200 plant species, representing over 50 families (Hayward 1994). It exhibits a strong tissue-specific tropism within the host, specifically invading and highly multiplying in the xylem vessels. In addition, it causes vascular browning, stunting, wilting and often leading to rapid death (Remenant et al. 2010). *R. solanacearum* is metabolically versatile and survives not only in soil but also in latently infected plants and water. The pathogen enters the plant through the roots (Xue et al. 2011). Thus, reliable methods to detect the pathogen in tubers as also in soil and soil-related habitats are required. *Xanthomonas perforans* is an important bacterial pathogen of vegetables particularly in tomato (*Lycopersicon esculentum* Mill.), causing serious economic losses worldwide (Kuflom et al. 1997). Bacterial spot disease of tomato occurs in warm, moist regions throughout the world. *X. perforans* is a rod-shaped Gram-negative aerobic bacterial plant pathogen. This spot pathogen is seed-borne, persist as epiphytic populations in asymptomatic seedling and mature plants. The bacterium possesses a polar flagellum that propels them in water, which facilitates the infection of wet leaves. The primary symptoms are necrotic lesions that occur on leaves, stem and fruits. In warm and rainy weather, bacterial spot may cause severe defoliation of plants that result in reduced yield, and the diseased fruits are not suitable for fresh-market sale. Blister-like fruit lesions and stem lesions are observed in late summer. *X. perforans* can survive in dry seeds for 10 years and on plants debris redundancy up to 1 year (Isabelle et al. 2006). The impact of both the pathogens in India and world over is very high.

The most commonly used methods are not accurate for identification of disease on visual symptoms. Isolation of pathogens from plants as well as seeds by conventional techniques is often difficult. Additionally, biochemical tests, pathogenicity test and serological tests take several weeks for the final confirmation of pathogens. The sensitivity of ELISA is limited only for the detection of symptomatic plants; enrichment of target bacteria on semi-selective media before ELISA improves specificity, but it is time consuming. Therefore, there is a need for rapid and specific method for routine indexing of asymptomatic and symptomatic plant materials (Lang et al. 2010).

Diagnostic tests based on the characteristics of the genomics of the bacterial pathogens can provide more reliable results, and these are not dependent on symptom expression and environmental conditions. An early DNA-based approach to distinguishing *R. solanacearum* and *X. perforans* involved amplification of 16S rRNA; the approach could be useful only if supported by other sequence information such as 16S–23S rRNA internal transcribed spacers, which is able to distinguish both pathogens (Schonfeld et al. 2003; Santana et al. 2012). Species-specific PCR-based methods are commonly used in the detection and identification of pathogens with the help of species-specific primers (Adachi and Oku 2000). A multiplex PCR amplification provides reliable pathogen detection in routine testing and allows for the simultaneous amplification of more than one DNA region of interest, which is possible in a single PCR reaction mixture.

Multiplex PCR is widely applied in simultaneous, rapid, accurate detection and identification of major pathogens having different DNA or RNA targets in a single reaction. Co-amplifications of *X. axonopodis* pv. *vesicatoria* and *Clavibacter michiganensis* sub spp*. michiganensis* were performed in tomato plant from a single PCR tube (Ozdemir 2005). A multiplex PCR assay targeting *avrBsT* and *xopL* for the molecular identification of *Xanthomonas axonopodis* pv. *phaseoli* was validated by comparison with other molecular identification assays aimed at *X. axonopodis* pv. *phaseoli*, on a wide collection of reference strains. This multiplex was further validated on a blind collection of *Xanthomonas* isolates for which pathogenicity was assayed by stem wounding and by dipping leaves in calibrated inoculation (Boureau et al. 2013).

Identification of *R. solanacearum* and *X. perforans* is an important step, as both pathogens have been considered as potential disease threat agents all over the world. Therefore, the primary objective of the present study was to develop a multiplex PCR, specific to *R. solanacearum* and *X. perforans* for identification of pathogen in symptomatic and asymptomatic samples.

## Materials and methods

### Bacterial isolation

Different cultivars of tomato seed samples were collected from the public and private seed traders, Mysore, India and agriculture fields Mysore, India. Suspected plant material viz, bacterial wilt and bacterial spot symptoms along with suspected soil samples from agriculture fields was collected, brought to the laboratory and subjected to laboratory assays viz, direct plating and liquid assay methods (ISTA 2005). The suspected plant material was cut into pieces (5 mm); plant materials as well as seeds were surface disinfected with sodium hypochlorite (3 %) followed by repeated washing with sterile distilled water and plated on semi-selective medium [Kelman’s triphenyl tetrazolium chloride (TZC; dextrose; 10 g, tryptone; 1 g, peptone; 10 g, agar; 18 g, dis H_2_O; 1,000 ml: triphenyl tetrazolium chloride; 0.075 g in 7.5 ml)] [Tween B media (peptone; 10 g, KBr; 10 g, CaCl_2_; 0. 25 g, boric acid; 0. 30 g, agar; 15 g, Tween 80; 10 ml, dis H_2_O; 1,000 ml)]. Following surface-sterilization, liquid assay of the collected seeds was carried out in plant material and seed samples were macerated using sterile mortar and pestle in 10 ml sterile distilled water. The supernatant (1 ml) was mixed with 9 ml of sterile distilled water to obtain a dilution of 10^−1^, and further serial dilutions were prepared up to 10^−5^. Fifty microliters of each dilution was placed on TZC and Tween B semi-selective media. In addition, the suspected soil samples from different fields were subjected to serial dilution up to 10^−5^ dilution, and aliquots of fifty microliters of each dilution were spread on TZC and Tween B semi-selective media using Drigalski’s spreaders in triplicates. Plates were incubated at 28 ± 2 °C for 24–48 h. The yellow colonies with hydrolytic zones were observed for *X. perforans* around the pieces of plant material and seeds, whereas typical mucoid creamy white colonies with pink centers were indicative for the presence of *R. solanacearum* (Hayward 1994). For positive control of *R. solanacearum* (DOBCPR 12), a pure culture used was kindly provided by Prof. Ashok Gadewar, Central Potato Research Institute, Shimla, India (Vanitha et al. 2009). These bacteria were isolated in their pure form and subjected to biochemical/physiological, hypersensitive and pathogenicity tests for confirmation of pathogens.

### Biochemical characterization of bacterial isolates

Biochemical characterization of *R. solanacearum* and *X. perforans* was performed based on biochemical tests: Gram staining, KOH solubility, starch hydrolysis test (Fahy and Persley 1983), lipase activity, Kovacs’ oxidase test (Kovac’s 1956), gelatin hydrolysis and oxidative/fermentative metabolism of glucose.

### Pathogenicity assay and hypersensitivity tests

Pathogenicity of bacterial isolates was assessed using two distinct tests. First, the virulence of *R. solanacearum* was tested with a pathogenicity assay on susceptible plants of tomato under the screen house conditions at 28 ± 2 °C. Susceptible cultivars of tomato (Cv. PKM-I) were sown in earthen pots, plants were allowed to grow for 5 weeks and used for bacterial inoculation. *R. solanacearum,* DOB-R strain was cultured in nutrient broth for 24–36 h. The density of cell suspension was adjusted to 1 × 10^8^ cfu/ml using spectrophotometer (Beckman Coulter, California, USA) (Lelliot and Stead 1987). This bacterial suspension was inoculated to the roots of tomato plants just below the soil surface.

*Xanthomonas perforans,* DOB-X strain suspension (1x10^8^ cfu/ml) was sprayed to the aerial parts of the tomato plants to completely run-off level, and plants were covered with polythene bags sprayed with water to maintain a high-humid condition. Pathogen-inoculated plants were closely monitored for the typical symptoms of both bacterial wilt and spot disease. Experiments were conducted in three replicates and repeated twice with appropriate controls.

Two milliliter of each bacterial suspension (1 × 10^8^ cfu/ml) was infiltrated to the leaves of 1 month old tobacco plant (*Nicotiana tabacum* L.) for hypersensitivity tests, with water as a controls (Carlton et al. 1998). Infiltrated tobacco plants were maintained under green house conditions with 25–30 °C in day and 15–18 °C at night and were maintained until the symptoms appeared.

### Specific PCR assay and sequence analysis

Bacterial isolates from soil, seed and plant material were used for the extraction of genomic DNA from *R. solanacearum* and *X. perforans.* Bacterial DNA was isolated using bacterial genomic DNA isolation kit from BangaloreGeNei (Bangalore, India) according to the manufacturer’s instruction. For identification of *R. solanacearum,* uniplex PCR was performed to amplify *speI* region of *R. solanacearum* using RS-F-759 and RS-R-760 primers as described by Opina et al. (1997). The specific primers RS-F-759 and RS-R-760 were custom synthesized from Chromous Biotech, Bangalore, India, RS-F (5′-GTCGCCGTCAACTCACTTTCC-3′) and RS-R (5′-GTCGCCGTCAGCAATGCGGAATCG-3′). DNA was amplified in 25 μl of reaction mixture prepared in 0.2-ml PCR tubes by adding PCR reaction mixture containing 1 μl of 100 mM dNTPs, 2.5 μl of 10× buffer, 2.0 μl of 25 mM MgCl_2_, 1 U of *Taq* DNA polymerase (Chromous Biotech, Bangalore, India), 1 µl of 10–100 ng genomic DNA and 2.0 μl of each primers of 25 pmol of *R.solanacearum* in a total volume of 25 μl. The PCR tubes were placed in a PCR thermocycler (Labnet, Multigene gradient, California, USA) and programmed thermal cycle as initial denaturation at 94 °C for 3 min, annealing at 53 °C for 1 min and extension at 72 °C for 1 min 30 s, followed by 30 cycles of denaturation at 94 °C for 15 s, annealing at 60 °C for 15 s, elongation step at 72 °C for 15 s and final extension at 72 °C for 5 min.

The isolates of *X. perforans* were subjected to PCR using specific primer which was selected at *hrp B* region of *Xanthomonas campestris* pv. *vesicatoria* strain 75-3 as stated by Leite et al. (1994) and custom synthesized as follows (RST2-5′AGGCCCTGGAAGGTGCCCTGGA3′) and (RST3-5′-ATCGCACTGCGTACCGCGCGCGA 3′). The PCR reaction mixture contained 1 μl of 100 mM dNTPs, 2.5 μl of 10× buffer, 2.0 μl of 25 mM MgCl_2_, 1 U of *Taq* polymerase (Chromous Biotech, Bangalore, India), 1 µl of 10–100 ng genomic DNA of *X. perforans,* 2.0 μl of each primers 25 pmol in a total volume of 25 μl. The PCR parameters were initial denaturing at 94 °C for 3 min, followed by 30 cycles at 94 °C for 30 s, 62 °C for 30 s, and 72 °C for 1 min 30 s, and a final extension at 72 °C for 7 min.

Amplified PCR products were purified using QIAquick gel extraction kit (Qiagen, Hilden, Germany) following the manufacturer’s instructions and sequenced commercially (Eurofins, Bangalore, India), and sequences were compared with other *R. solanacearum and X. perforans* sequence from data base using multiple sequence alignment software. Sequences of both pathogens were deposited in the GenBank database.

### Optimization of multiplex PCR

Multiplex PCR was carried out for bacterial DNA templates isolated from mixed culture of both pathogens. A primer mix was used with final concentration of 100 pmol of each primer in a ratio of 1:4, which was standardized among 1:1, 1:2, 1:3 and 1:4 for *R. solanacearum* and *X. perforans*, respectively. Reactions in a total volume of 25 µl were performed with 1 U of *Taq* DNA polymerase (Chromus Biotech, Bangalore, India). DNA was amplified in 25 μl of reaction mixture containing 10× thermo pol buffer, 2 mM MgCl_2_, and 25 mM of dATP, dCTP, dGTP and dTTP. The cycling was performed with a Master cycler Gradient (Labnet, Multigene gradient, California, USA). Gradient PCR was performed to optimize the multiplex PCR annealing temperature from 55 to 65 °C. The PCR parameters were followed an initial denaturing step at 95 °C for 3 min, followed by 30 cycles of 95 °C for 30 s, 61 ± 1 °C for 1 min and 72 °C for 1 min. The amplified PCR product was mixed with 2 µl of loading dye and separated in 1.2 % agarose gel electrophoresis by TAE buffer using 75 V. Further, gels were documented using Geldoc 1000 System-PC (Bio-Rad, Gurgaon, India). *C. michiganensis* subsp. *michiganensis* and *Pseudomonas fluorescens* were used as negative controls (Table 1).

**Table 1:** Mixed culture, isolation of *R. solanacearum* and *X. perforans* from different sources and their reaction to multiplex PCR

Source	Isolates of *R. solanacearum*	Isolates of *X. perforans*	Multiplex PCR	
			*R. solanacearum*	*X. perforans*
Soil	RS 1	XP 1	+	+
Soil	RS 2	XP 2	+	+
Soil	RS 3	XP 3	+	+
Soil	RS 4	XP 4	+	+
Soil	RS 5	XP 5	+	+
Plant material	RS 6	XP 6	+	+
Plant material	RS 7	XP 7	+	+
Plant material	RS 8	XP 8	+	+
Plant material	RS 9	XP 9	+	+
Plant material	RS 10	XP 10	+	+
Seed	RS 11	XP 11	+	+
Seed	RS 12	XP 12	+	+
Seed	RS 13	XP 13	+	+
Seed	RS 14	XP 14	+	+
Seed	RS 15	XP 15	+	+
Seed	RS 16	XP 16	+	+
Seed	RS 17	XP 17	+	+
Seed	RS 18	XP 18	+	+
Seed	RS 19	XP 19	+	+
Seed	RS 20	XP 20	+	+
Seed	Cmm	Cmm	–	–
Seed	PS	PS	–	–

RS 1–RS 5. Bacterial isolates isolated from serial dilution method, RS 6–RS 10. Bacterial isolates isolated from direct plating method of plant materials (stem), RS 11–Rs 15. Bacterial isolates isolated from direct plating method of seeds, RS 16–RS 20. Bacterial isolates isolated from liquid assay method for seeds

XP 1–XP 5. Bacterial isolates isolated from serial dilution method, XP 6–XP 10. Bacterial isolates isolated from direct plating method of plant materials (leaves), XP 11–XP 15. Bacterial isolates isolated from direct plating method of seeds, XP16–XP 20. Bacterial isolates isolated from liquid assay method for seeds

Cmm. *Clavibacter michiganensis* subsp. *michiganensis* and PS. *Pseudomonas fluorescens* bacterial isolate isolated from direct plating method of seed and soil, respectively

### Validation of multiplex PCR

Developed multiplex PCR was validated by artificially infecting susceptible tomato plants with DOB-R and DOB-X strains, respectively, with 20 different cultivars (five plants with four replicates) (Table 2), along with infected plants collected from agricultural fields. The seed samples were macerated using sterile mortar and pestle in 1 ml sterile distilled water, and soil samples were serially diluted up to 10^−3^, and 10 µl of macerated seed sample and diluted soil samples were used as template to amplify multiplex PCR. The sensitivity of the primers was checked by diluting single-colony bacterial cultures from 10^−1^ up to 10^−5^ in sterile distilled water (100 µl in 900 µl of sterile distilled water) without isolating DNA from the bacterial cultures in this multiplex PCR, and gel was documented using Geldoc 1000 System-PC (Bio-Rad, Gurgaon, India).

**Table 2:** Mixed infection of pathogens in susceptible plants compared by pathogenicity test and multiplex PCR

Bacterial isolates	Tomato cultivars	Response to pathogenicity test		Response to multiplex PCR	
		*R. solanacearum*	*X. perforans*	*R. solanacearum*	*X. perforans*
1	Ark-Abha	+	+	+	+
2	Ashwini-FI	+	+	+	+
3	Arunodaya	+	+	+	+
4	Indosem	+	+	+	+
5	Indam	+	+	+	+
6	Madanapalli	+	+	+	+
7	Malini	+	+	+	+
8	MPH-I	+	+	+	+
9	OK Seed	+	+	+	+
10	PKM-I	+	+	+	+
11	Sarapana	+	+	+	+
12	HCl-IV	+	−	+	−
13	Local-I	+	−	+	+
14	PHS	+	−	+	+
15	Ashoka	−	−	+	+
16	Alrounder	−	−	+	+
17	Rasi	−	+	−	−
18	Quality	−	+	−	−
19	Vignesh	−	−	+	−
20	Mrytunjaya	−	−	−	+

“+” indicates the positive reaction; “−” indicates negative reaction of phytopathogenic bacteria

## Results and discussion

The bacterial wilt-causing pathogen *R. solanacearum* and bacterial spot-causing pathogen *X. perforans* in tomato plants were isolated and characterized. Plant material, seed samples and soil samples were subjected to laboratory assays such as direct plating and liquid assay. The samples showed the presence of both the pathogens. Isolates of *R. solanacearum* from soil, plant material and seeds were cultured using semi-selective media, and typical mucoid creamy white colonies with pink centers were observed. *X. perforans* colonies on Tween B media exhibited typical morphological characteristics such as yellow colonies with hydrolytic zones. Further, different isolates of *R. solanacearum* and *X. perforans* were subjected to biochemical/physiological assays along with hypersensitivity and pathogenicity tests. Phytobacterial pathogens, *C. michiganensis* subsp. *michiganensis* and *P. fluorescens* were also isolated from the collected seed and soil samples, respectively (Table 1).

*R. solanacearum* and *X. perforans* were subjected to biochemical characterization, both pathogens stained pink red for Gram’s reaction and thin viscid mucoid strand for KOH solubility indicating Gram’s negative in nature. *X. perforans* liquefied gelatin media when compared control and show a clear zone of hydrolysis around the bacterial colonies when flooded with Lugol’s iodine on starch hydrolysis’ test. Lypolytic activity was confirmed positive by the presence of a white precipitate around the colonies of *X. perforans* in Tween 80 agar plates. *R. solanacearum* designate negative for gelatin hydrolysis, starch hydrolysis test as well as lipase activity tests. Kovac’s oxidase tests show a positive result by immediate change in the color to blue in *R. solanacearum,* but it was negative in *X. perforans. R. solanacearum* changed the color of media from green to yellow, indicating positive results for oxidation test, whereas in the fermentation test, both these pathogens did not show any reaction.

Susceptible tomato cultivars when inoculated with *R. solanacearum* isolated from the soil, seed and plant material showed bacterial wilt symptoms such as vascular browning, stunting, wilting and often with rapid death of tomato plants. Control plants did not show any disease symptoms. *X. perforans* isolates showed bacterial spot symptoms, whereas control plants with nutrient broth did not show any symptoms. Yellowing of leaf followed by necrosis was evident in tobacco plants within 48 h of infiltration with both bacterial isolates, whereas control leaves did not show any change in leaf morphology. Biochemical/physiological tests, hypersensitivity and pathogenicity tests were used in the identification and confirmation of the isolated pathogens as *R. solanacearum* and *X. perforans*, but these tests are time consuming. Our main objective was to develop a reliable multiplex PCR assay for the identification of these pathogens. This new PCR assay combines two different tests first, high specificity in the identification of the pathogens. The results of present study are in confirmation with the studies reported, earlier (Chandrashekar et al. 2012; Avinash and Umesha 2014).

Polymerase chain reaction technique has found wide application in detecting plant pathogenic bacteria (Adachi and Oku 2000). Targeting 16S–23S spacer region is not specific, but it is also fast and easy. A multiplex “Taq man” PCR method was described by Weller et al. (2000) for specific identification of *R. solanacearum* in potato tubers extract, the developed multiplex PCR described here is a simple, with lower cost and an alternative to the “Taq man” method. A multiplex PCR method for detection and differentiation of *R. solanacearum* strains compared with 16S–23S rRNA primers with the use of sequence analysis which differentiate *R. solanacearum* subclasses which is also cost effective for large-scale screening. The 16S–23S spacer region has large copy number (more than 10,000 per cell) and high degree of sequence conservation, which are the drawbacks of 16S–23S primers Pastrik et al. (2002).

*R. solanacearum* subjected to specific PCR assay using specific primers viz, RS-F-759 and RS-R-760 showed a specific amplification at 281 bp, confirmed as *R. solanacearum*. Primers RST2 and RST3 showed an amplification of 840-bp amplicon in genome confirmed as *X. perforans*. To confirm primer specificity, homology test for primers was carried out using BLAST search considering query coverage and percentage identity for both pathogens, in which RS-F-759 and RS-R-760 primers showed 100, 95 and 75 % homology to *R. solanacearum, R. syzygii* and *Oryza minuta*, respectively, whereas same primers did not show homology with *Xanthomonas*. The *hrp B* primers did not show 100 % identity with *X. oryzae* pv. *oryzae*, *X. oryzae* pv. *oryzicola* and *X. fuscans* subsp. *fuscans*, whereas *hrp B* primers did not show homology with *R. solanacearum*. Identities of pathogens were further confirmed by sequencing of amplified products of *R. solanacearum* and *X. perforans*, and sequences were deposited in the GenBank database (accession numbers JX628912 and JX628913). To examine further the extant of sequence variation present in *R. solanacearum* and *X. perforans,* nucleotide sequence of other strains of both pathogens from GenBank database was compared. The result revealed that 100 % homology with other *R. solanacearum* strains, whereas *X. perforans* sequence showed partial similarity with *X. oryzae* pv. *oryzae,* but it showed a non-specific amplification when cross-tested with primers RST2 and RST3.

**Fig. 1:**
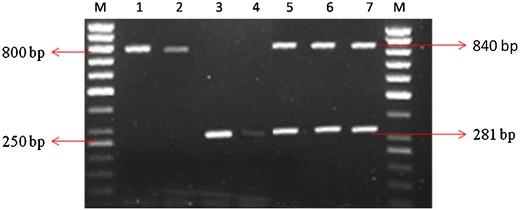
Optimized multiplex PCR for both *R. solanacearum* and *X. Perforans. Lanes 1* and *2* amplification of *R. solanacearum* annealing temperature at 57 and 61 °C. *Lanes 3* and *4* amplification of *X. perforans* annealing temperature at 58 and 64 °C. *Lanes 5*–*7* multiplex PCR of *R. solanacearum* and *X. perforans* 61 ± 1 °C. 50 bp (*M*) Gene ladder

**Fig. 2:**
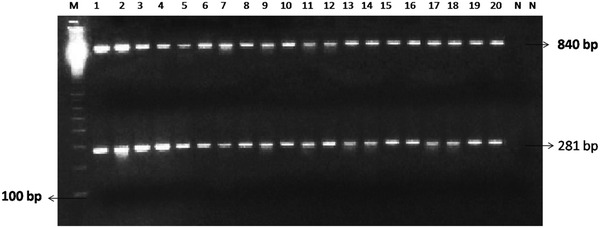
Multiplex PCR for both *R. solanacearum* and *X. perforans.Lanes 1*–*20* mixed culture of *R. solanacearum* and *X. Perforans* amplified at 281 and 840 bp. (N) Negative control *Clavibacter michiganensis* subsp. *michiganensis* and *Pseudomonas fluorescens*. 100-bp DNA marker (*M*)

A single gradient multiplex PCR was used to detect *R. solanacearum* and *X. perforans* by determining the range of annealing temperature. The gradient multiplex PCR was carried out using annealing temperature from 55 to 65 °C, PCR amplified an amplicon of 281 bp for *R. solanacearum* in gradient PCR, whereas the intensity of bands was decreased continuously at increasing the annealing temperature from 57 to 61 °C and amplifications were not observed above 61 °C for *R. solanacearum. X. perforans* showed decreased intensity of the band for annealing temperature range from 57 to 61 °C, whereas amplifications were observed at 840-bp amplicon at 61 °C, thereby standardizing the concentration of DNA and concentration of primers efficacy with respect to *X. perforans.* In primer concentration of 1:4, *R. solanacearum* and *X. perforans*, respectively, showed optimum amplifications at the annealing temperature 61 ± 1 °C (Fig. 1). *C. michiganensis* subsp. *michiganensis* and *P. fluorescens* did not show any amplification in multiplex PCR. All isolates collected from field conditions viz, seed samples, plant material along with soil samples (Table 1) amplified in a multiplex PCR simultaneously which confirms both pathogens as *R. solanacearum* and *X. perforans* (Fig. 2). Earlier, a multiplex PCR assay was developed for simultaneous detection of *C. michiganensis* subsp. *michiganensis, Pseudomonas syringae* pv. *tomato* and *X. axonopodis* pv*. vesicatoria* (Ozdemir 2009). The present multiplex PCR technique developed could be a very useful approach for early identification of *R. solanacearum* and *X. perforans* in the current agriculture. On the basis of 23S rRNA gene sequences, one universal forward and four taxon-specific reverse primers were designed for multiplex PCR to aid in identification and differentiation of *Agrobacterium rubi, A. vitis* and *A.* biovars 1 and 2 (Puławska et al. 2006). The multiplex PCR can be a better tool for rapid classification of similar species and taxa of phytopathogenic bacteria which can be used to identify different biovars among *R. solanacearum* and *X. perforans*. The developed multiplex PCR is more efficient than a single PCR reaction in saving time and reduces reaction cost by simultaneous amplification of pathogens.

**Fig. 3:**
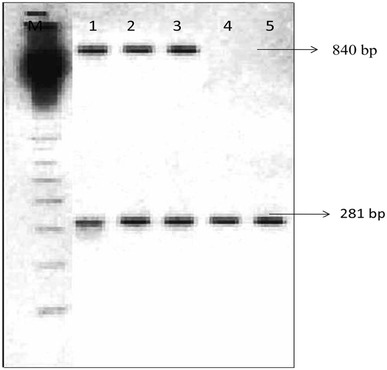
Multiplex PCR amplification of *R. solanacearum* and *X. perforans* from different soil samples. Amplification at 840 bp indicates *X. perforans*, and amplification at 281 bp indicates *R. solanacearum* 100-bp DNA (*M*)

Twenty different tomato cultivars (five plants in four replicates) were artificially inoculated with the isolates of *R. solanacearum* and *X. perforans* to validate multiplex PCR (Table 2). Leaves were collected from inoculated plants after 8–10 days, and pathogens were isolated by plating the leaves on nutrient agar media. In addition, multiplex PCR performed to identify *R. solanacearum* and *X. perforans* from single-colony PCR to increase sensitivity of amplification. Both the pathogens amplified up to 10^−3^ dilution, indicating that the developed method is useful for identification of both the phytobacterial pathogens without DNA isolation (Fig. 2), correspondingly seed and soil samples which also showed amplification in 10^−2^ dilution which validate the developed multiplex is extremely valuable in early diagnosis of pathogen from seed, soil (Fig. 3) as well as plant materials. The number of contaminations within a system may be low, thereby making it difficult to detect infested soil, plant material and seeds. To tackle these issues, a multiplex PCR technique was developed in the present study is of great value to distinguish *R. solanacearum* and *X. perforans*. The multiplex PCR was found to be a better technique as it could detect more positive-infected seed samples when compared to the conventional method. The pathogenicity test conducted in susceptible plants of tomato isolates 12–16 exhibited negative results, whereas it showed positive results in multiplex PCR. Interestingly, the isolate 17 (Rasi) gave positive for pathogenicity, but negative in multiplex PCR which overcomes the false positive results (Table 2). Multiplex PCR protocols have been developed to detect several pathogens or genetically heterogeneous strains of a single pathovar simultaneously. Even detection of one bacterium and four viruses was reported by multiplex PCR in olive plants (Bertolini et al. 2003). The early diagnosis of bacterial wilt and spot diseases in this crop is very essential in current scenario to develop suitable management strategies which lead to improvement in the yield of agricultural products.

## Conclusion

It can be concluded that the multiplex PCR technique described in the present study is a reliable, sensitive and cost effective procedure for identifying *R. solanacearum* and *X. perforans.* The present technique developed is useful for international sanitary surveillance of planting material exchanges. Additionally, this molecular tool can be useful to determine the relationship between soil/seed contamination and disease incidence of *R. solanacearum* and *X. perforans* in tomato. Furthermore, this method can assess the relative importance of different stages of dissemination of these pathogens.
